# Cancer-Associated Fibroblast (CAF) Heterogeneity and Targeting Therapy of CAFs in Pancreatic Cancer

**DOI:** 10.3389/fcell.2021.655152

**Published:** 2021-07-15

**Authors:** Xinglong Geng, Hongze Chen, Liang Zhao, Jisheng Hu, Wenbo Yang, Guanqun Li, Chundong Cheng, Zhongjie Zhao, Tao Zhang, Le Li, Bei Sun

**Affiliations:** ^1^Department of Pancreatic and Biliary Surgery, The First Affiliated Hospital of Harbin Medical University, Harbin, China; ^2^Key Laboratory of Hepatosplenic Surgery, Ministry of Education, The First Affiliated Hospital of Harbin Medical University, Harbin, China; ^3^Department of Abdominal Endoscopic Surgery, Affiliated Hospital of Qinghai University, Xining, China; ^4^Department of Gynecology, Qinghai University Affiliated Hospital, Xining, China

**Keywords:** cancer-associated fibroblasts, heterogeneity, pancreatic cancer, hallmark, diagnosis, prognosis, therapy

## Abstract

Pancreatic ductal adenocarcinoma (PDAC) is a highly lethal disease that typically features a dramatic desmoplastic reaction, especially fibroblasts. The roles of cancer-associated fibroblasts (CAFs) in PDAC have received more attention in recent years. As increasing evidence suggests the heterogeneity of CAFs in PDAC, different CAF subtypes have been shown to support tumor growth, while others suppress cancer proliferation. Myofibrotic CAFs (myCAFs) show alpha-smooth muscle actin (α-SMA)^*high*^ interleukin-6 (IL-6)^*low*^ myofibroblastic features, are activated by direct contact with tumor cells, and are located in the periglandular region. Inflammatory CAFs (iCAFs) show α-SMA^*low*^ IL-6^*high*^ inflammatory features, are activated by paracrine factors secreted from tumor cells, and are located away from cancer cells. Antigen-presenting CAFs (apCAFs) show major histocompatibility complex II (MHC II) family genes that are highly expressed. CAFs have also been gradually explored as diagnostic and prognostic markers in pancreatic cancer. Targeted therapy of CAFs in PDAC has gradually attracted attention. With the deepening of related studies, some meaningful positive and negative results have surfaced, and CAFs may be the key to unlocking the door to pancreatic cancer treatment. Our review summarizes recent advances in the heterogeneity, function, and markers of CAFs in pancreatic cancer, as well as research and treatment targeting CAFs in pancreatic cancer.

## Introduction

Pancreatic ductal adenocarcinoma (PDAC) is a highly aggressive malignant cancer with the 12th highest morbidity and the 4th highest mortality worldwide (Siegel et al., 2020). The feature of PDAC is a dramatic desmoplastic reaction, including fibroblasts, macrophage, immune cells, and a dense extracellular matrix (ECM) (Feig et al., 2012). Recently, the Banbury Center meeting convened experts to discuss cancer-associated fibroblast (CAF) biology and issue a consensus statement; they view that cells negative for epithelial, endothelial, and leukocyte markers with an elongated morphology and lacking the mutations found within cancer cells might be considered CAFs (Sahai et al., 2020). CAFs could remodel the ECM (Gaggioli et al., 2007; Goetz et al., 2011) and decrease CD36 expression (DeFilippis et al., 2012). CAFs could interfere with drug delivery (Jacobetz et al., 2013) and produce collagen in the extracellular space to modulate tumor stiffness and facilitate tumor progression (Olivares et al., 2017). CAFs can secrete chemokines (Scherz-Shouval et al., 2014; Kumar et al., 2017) and cytokines (Biffi et al., 2019), which leads to lymphatic angiogenesis (Cadamuro et al., 2019) to promote cancer cell intravasation. They also alter the immune cell milieu by recruiting immune suppressive cells and inhibiting the activity of immune effector cells (Öhlund et al., 2017). It is believed that if the expression of CAFs can be inhibited, the differentiation of tumor cells can be inhibited. Depleting alpha-smooth muscle actin (α-SMA)-expressing cells or inhibiting sonic hedgehog (Shh), a ligand that could activate CAFs, induced immunosuppression, undifferentiated tumors, and decreased survival in mice (Kim et al., 2014; Lee et al., 2014; Rhim et al., 2014; Özdemir et al., 2014). Increasing data suggest heterogeneity among CAFs; some CAF subtypes promote tumor growth, and some suppress tumor growth. In addition, many studies on stroma in other tumors have also found and proposed the heterogeneity of CAFs. For example, Costa et al. (2018) identified four subsets of CAFs in breast cancer. Among them, CAF subset 1 causes an immunosuppressive microenvironment by inhibiting CD4^+^CD25^+^ T cells. Su et al. (2018) found that CD10^+^GPR77^+^ CAFs, as a new subset, could promote tumor formation in breast and lung cancer patients. Due to heterogeneity, CAFs have different markers, sources, nomenclatures, functions, and even mechanisms. Therefore, our review aims to demonstrate the heterogeneity of CAFs in PDAC and summarize the current heterogeneity of CAFs related to PDAC and the prognosis, diagnosis, and therapy related to CAFs.

## Heterogeneity of CAFs in PDAC

The heterogeneity of CAFs was raised at an early stage. Ikenaga et al. (2010) showed two subtypes of CAFs in human PDAC by flow cytometry. Baron et al. (2016) used single-cell RNA sequencing (scRNA-seq) to examine more than 12,000 pancreatic cells and identified three types of human pancreatic stellate cells (PSCs) and the existence of two modes of PSC activation. Activated PSCs are known as CAFs (Bachem et al., 2005). Öhlund et al. (2017) established a 3D coculture system to recapitulate the desmoplastic reaction of PDAC *in vitro* and found two distinct subpopulations of CAFs, myofibroblastic CAFs (myCAFs), and inflammatory CAFs (iCAFs). Later, Elyada et al. (2019) applied scRNA-seq to PDAC tissue and further reconfirmed myCAFs and iCAFs. Surprisingly, they even found a third CAF subtype, named antigen-presenting CAFs (apCAFs). Dominguez et al. (2020) and Hosein et al. (2019) confirmed the presence of myCAFs, iCAFs, and apCAFs in mice by scRNA-seq, consistent with a previous description. Bernard et al. (2019) confirmed the presence of myCAFs and iCAFs in human PDAC tissues *via* scRNA-seq. Exploring the contact between tumors and CAFs is an important direction of future research and may even be an important therapeutic strategy. We then summarize the three types of CAFs.

### myCAFs

Öhlund et al. (2017) conducted a unique study on CAFs. First, they analyzed human PDAC fibroblasts using fibroblast-activation protein (FAP) and α-SMA by immunofluorescence, and the results showed that most fibroblasts expressed FAP and low levels of α-SMA, whereas a small fraction expressed FAP and high levels of α-SMA. These results confirmed the heterogeneity hypothesis. Next, they invented a novel 3D coculture system that combined tumor-derived organoids with PSCs. They found that a subtype of CAFs with high expression of α-SMA and low expression of IL6 was activated only when tumor cells came into direct contact with PSCs, and they defined those CAFs as myCAFs. Meanwhile, myCAFs were observed in the periglandular region *in vivo*, again verifying that the formation of myCAFs requires direct interaction with cancer cells in PDAC. Otherwise, connective tissue growth factor (Ctgf) and collagen type I alpha 1 (Col1α1), which respond to actin alpha 2 (Acta2) and transforming growth factor-β (TGF-β), were upregulated in myCAFs (Öhlund et al., 2017). Elyada et al. (2019) reconfirmed the presence of myCAFs through sRNA-seq and identified a cluster of new marker genes, including transgelin (TAGLN), myosin regulatory light chain 9 (MYL9), and tropomyosin 1 (TPM1). The pathways enriched in myCAFs were identified by gene set enrichment analysis (GSEA), including smooth muscle contraction, focal adhesion, ECM organization, and collagen formation. Protein activity analysis showed activated proteins in myCAFs, including TGF-β1, SMAD family member 2 (SMAD2), and Twist family BHLH transcription factor 1 (TWIST1) (Elyada et al., 2019). Biffi et al. (2019) found that TGF-β promotes fibroblast transformation to myCAFs and inhibits fibroblast transformation to iCAFs by downregulating IL1R1 expression. The TGF-β/SMAD2/3 pathways could induce myCAF activation. They believed that myCAFs and iCAFs are interchangeable, depending on their location and exposure in the tumor (Biffi et al., 2019). Interestingly, Bernard et al. (2019) found that myCAFs were rare in low-grade IPMNs but were highly represented in high-grade IPMNs, suggesting that myCAFs could occur in non-invasive neoplasia. Sun et al. (2021) showed that chemokine (C-X-C motif) ligand 3 (CXCL3)-C-X-C motif chemokine receptor 2 (CXCR2) signaling converted CAFs into myCAFs, which hijacked low metastatic cancer cells for metastasis. They proposed that the “soil-carry-seed” concept for PDAC metastasis meant myofibroblasts (soil) hijack cancer cells (seed) for metastasis (Sun et al., 2021). A study revealed that fibroblasts switch into 15-leucine repeat membrane protein (LRRC15)^+^ myCAFs as a determinant of patient response to cancer immunotherapy, which was associated with poor clinical response (Dominguez et al., 2020). Recently, Chen et al. (2021) revealed that the deletion of Col1 in α-SMA^+^ myofibroblasts using mouse models decreases stromal Col1 content and accelerates the progression of pancreatic cancer. In summary, we conclude that myCAFs show α-SMA^*high*^ interleukin-6 (IL-6)^*low*^ myofibroblastic features, activated by direct contact with tumor cells, and are located in the periglandular region. The TGF-β/SMAD2/3 pathway could induce myCAF activation in PDAC. myCAFs are related to metastasis and immunosuppression in PDAC. At present, further experimental studies are needed to confirm whether these two cellular states can be converted into each other *in vivo* and the effect of promoting the occurrence and development of pancreatic tumors. We may build a target to intervene in the treatment of pancreatic cancer to improve the prognosis of pancreatic cancer.

### iCAFs

In the 3D coculture system, Öhlund et al. (2017) found a subtype of CAFs with α-SMA^*low*^ IL-6^*high*^ inflammatory features and loss of myofibroblastic features. The subtype of CAFs was defined as iCAFs. iCAFs were activated by paracrine factors secreted from tumor cells, and their location was distant from tumor cells and myCAFs. They also revealed that IL-6, IL-11, CXCL1, CXCL2, and leukemia inhibitory factor (LIF) were particularly upregulated in iCAFs by scRNA-seq (Öhlund et al., 2017). Elyada et al. (2019) confirmed the presence of iCAFs through scRNA-seq in human and mouse tumors. They found that iCAFs upregulated new marker genes, such as IL6, IL8, and CXCL12. In addition, iCAFs specifically expressed hyaluronic acid synthase 1 (HAS1) and HAS2, two enzymes responsible for the synthesis of hyaluronic acid, which is considered to be an important barrier to the treatment of PDAC. They identified pathways enriched in iCAFs by GSEA, including interferon γ (IFN-γ) response, tumor necrosis factor (TNF)/nuclear factor kappa-B (NF-κB), IL2/signal transducer and activator of transcription (STAT) 5, and IL6/Janus kinase (JAK)/STAT3 in humans. To identify differentially activated proteins between iCAFs and myCAFs, protein-activity analysis was adopted, and IL1R1 and STAT3 were found to be differentially activated in iCAFs. Surprisingly, TGF-β receptors TGF-βR2 and TGF-βR3 were the top differentially activated proteins in iCAFs, which do not show an activated TGF-β program and may be caused by a loss of TGF signaling in iCAFs (Elyada et al., 2019). Biffi et al. (2019) proved that tumor-secreted IL-1 activated the LIF/JAK/STAT pathway to activate iCAFs in human and mouse PDAC and decreased myofibroblastic genes, including the connective Ctgf-TGF-β target gene Acta2-α-SMA gene. Shi et al. (2019) showed that LIF secreted by PSCs was associated with tumor progression in PDAC. Interestingly, Bernard et al. (2019) found that the iCAF population was abundant in PDAC but absent in low-grade and high-grade IPMN, which might show that iCAFs promote IPMN transformation into PDAC. Garg et al. (2018) found that iCAFs release CXCL12 in an NFκB-mediated manner, leading to immunosuppression and promoting tumor growth in PDAC. Above all, we conclude that iCAFs show α-SMA^*low*^ IL-6^*high*^ inflammatory features, are activated by paracrine factors from tumor cells, and are located away from cancer cells. The Il-1/LIF/JAK/STAT pathway could activate iCAFs, which suppress immune and ECM deposition and accelerate the occurrence and progression of PDAC. iCAFs are closely related to inflammation. Further studies on the relationship between inflammatory factors, iCAFs, and tumor cells can be conducted to determine whether the mechanism of pancreatic tumors can be discovered, and the inhibition of related factors combined with chemotherapy to improve the prognosis of pancreatic cancer is worth investigating in the future.

### apCAFs

**FIGURE 1:**
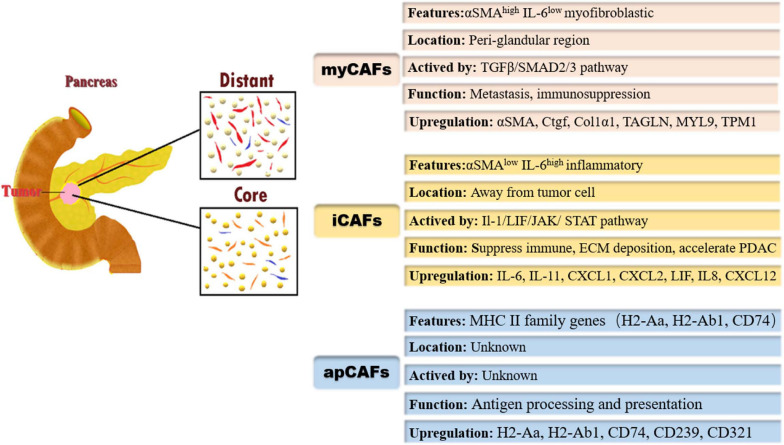
Heterogeneity of cancer-associated fibroblasts (CAFs) and their markers/pathways; the different subpopulations of CAFs are geographically segregated in PDAC tumor microenvironment. myCAFs, myofibroblastic cancer-associated fibroblasts; αSMA, α-smooth muscle actin; IL-6, interleukin-6; TGF-β, transforming growth factor-β; SMAD, *Drosophila* mothers against decapentaplegic protein; Ctgf, connective tissue growth factor; Col1α1, collagen type I alpha 1; TAGLN, transgelin; MYL9, myosin regulatory light chain 9; TPM1, tropomyosin 1; iCAFs, inflammatory cancer-associated fibroblasts; JAK, Janus kinase; STAT, signal transducer and activator of transcription; CXCL1, chemokine (C-X-C motif) 1; apCAFs, antigen-presenting cancer-associated fibroblasts; MHC II, major histocompatibility complex II; H2-Aa, encoding α-chains of MHC II; H2-Ab1, encoding β-chains of MHC II.

Elyada et al. (2019) identified a subtype of CAFs that expressed MHC class II family genes. The MHC II family is usually expressed in antigen-presenting cells; therefore, they named this group of fibroblasts “antigen-presenting CAFs” (apCAFs). apCAFs expressed unique genes, including H2-Aa (encoding α-chains of MHC II), H2-Ab1 (encoding β-chains of MHC II), CD74 (encoding invariant chains of MHC II), serum amyloid A3 (Saa3), and secretory leukocyte peptidase inhibitor (SLPI). The pathways enriched in apCAFs were identified, including antigen presentation and processing, fatty acid metabolism, MYC targets, and mTOR complex 1 (MTORC1) signaling. They found activated proteins in apCAFs, except the most highly activated proteins H2-Ab1 and CD74, which also included CD239, CD321, and interferon regulatory factor 5 (IRF5) (Elyada et al., 2019). Dominguez et al. (2020) also found that apCAFs highly expressed CD74, H2-Ab1, and Saa3. Hosein et al. (2019) proved that a subtype of CAFs could perform antigen processing and presentation through the MHC-II pathway and had complement activation functions. apCAFs could express costimulators such as professional antigen presenting cells (APCs), but their levels were significantly lower, indicating that apCAFs act differently from professional APCs in PDAC. In addition, apCAFs are regulated by IFN-γ signaling *in vivo* and exhibit an antioxidant response (Elyada et al., 2019). In summary, apCAFs show high expression of MHC II family genes; however, the activation conditions and their locations need to be further studied. There is a large space to explore the immune relationships between CAFs and the pancreatic tumor microenvironment and research on targeted drugs in PDAC (Figure 1).

## Hallmarks for Diagnosis and Prognosis of PDAC by CAFs

**FIGURE 2:**
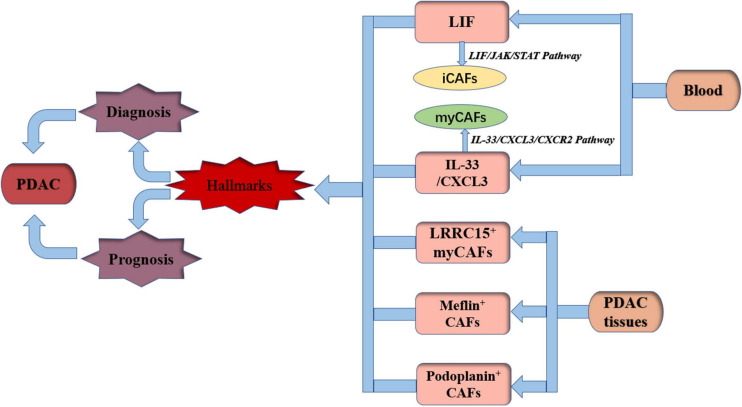
Hallmarks for diagnosis and prognosis of PDAC by CAFs. PDAC, pancreatic ductal adenocarcinoma; LIF, leukemia inhibitory factor; iCAFs, inflammatory cancer-associated fibroblasts; myCAFs, myofibroblastic cancer-associated fibroblasts; IL-33, interleukin-33; CXCL3, chemokine (C-X-C motif) 3; LRRC15, 15-leucine repeat membrane protein; JAK, Janus kinase; STAT, signal transducer and activator of transcription; CXCR2, C-X-C motif chemokine receptor 2.

Histological studies revealed that higher expression of stromal proportion or α-SMA is predictive for poor clinical outcome of PDAC patients (Erkan et al., 2008). However, depletion of α-SMA-expressing cells resulted in immunosuppression and decreased survival in mice (Özdemir et al., 2014). A specific CAF marker or a certain type of CAF plays a more important role in PDAC diagnosis and prognosis judgment. It has been found that LIF can activate CAFs independently of the expression of α-SMA (Albrengues et al., 2014). Biffi et al. (2019) identified that the IL1-induced JAK/STAT pathway promoted an inflammatory CAF state. Changes in circulating LIF levels were closely related to tumor response to treatment (Shi et al., 2019). Thus, LIF would be an attractive therapeutic target and circulatory marker. IL-33 is specifically elevated in human PDAC, and IL-33 stimulates the CXCL3-CXCR2 axis to convert CAFs into myCAFs, which hijack low metastatic cancer cells for metastasis (Sun et al., 2021). Sun et al. (2021) found that IL-33 and CXCL3 levels were inversely associated with survival in patients with PDAC. It can be inferred that IL-33 and CXCL3 can be used as prognostic markers in PDAC. Dominguez et al. (2020) found that LRRC15^+^ myCAFs surrounded tumor islets and were absent from normal tissue in PDAC. An immunotherapy clinical trial involving more than 600 patients showed that elevated levels of LRRC15^+^ myCAF signaling were associated with poor outcomes after anti-PD-L1 therapy (Dominguez et al., 2020). Consequently, LRRC15^+^ myCAFs could be a prognostic marker for immune checkpoint blockade therapy in PDAC. Mizutani et al. (2019) found that Meflin^+^ CAFs were associated with favorable outcomes using *in situ* hybridization of tissues from 71 PDAC patients. Thus, Meflin^+^ CAFs could be used as a marker of favorable prognosis for PDAC patients. In a meta-analysis of 4,000 patients from 29 studies, it was found that podoplanin^+^ CAFs resulted in worse survival rates for patients with solid tumors (Hu et al., 2018), suggesting that podoplanin^+^ CAFs are a valuable prognostic marker. In addition to the above studies, it is possible to evaluate the stage of PDAC disease by combining a number of markers to dynamically monitor the state and the interconversion of CAFs instead of taking a certain marker as the standard for judging prognosis to provide stronger and reliable evidence for treatment (Figure 2).

## Targeting Therapy of CAFs in PDAC

Blockade of programmed death 1 (PD-1)/programmed cell death-ligand 1 (PD-L1) and cytotoxic T-Lymphocyte associated protein 4 (CTLA-4) has improved the prognosis of some tumors (Hamid et al., 2013; Perez-Ruiz et al., 2019), but PDAC could not benefit from all of these treatments (Royal et al., 2010; Brahmer et al., 2012). Despite the presence of numerous immune cells, only immunosuppressive cells appear in the TME, and restraining effector immune cells enter the tumor stroma (Mu et al., 2019). The immunosuppressive effect of PDAC is related to CAFs. Therefore, targeting CAF therapy in PDAC may become an important direction of future research. Abundant CAFs could compress blood vessels and compromise the systemic delivery of therapeutic agents (Stylianopoulos et al., 2012). CAFs also entrapped active therapeutic drugs inside cells, limiting drug delivery to tumor cells (Hessmann et al., 2018). CAFs can remodel the ECM to enhance stromal stiffening and promote tumor invasion (Calvo et al., 2013). Exosomes secreted by CAFs could lead to chemoresistance (Hu et al., 2015). CAFs could directly inhibit the antitumor T cell response and improve the survival of tumors (Lakins et al., 2018). CAFs in PDAC were highly expressed with β-Ig-H3 protein, which could directly inhibit tumor-specific CD8^+^ T cells, leading to immunosuppression and tumor growth (Goehrig et al., 2019). Stromal cell-derived factor-1 (SDF-1) secreted by CAFs can promote tumor malignancy and gemcitabine resistance in PDAC (Wei et al., 2018). Decreased levels of CD146 in CAFs promote tumor progression (Zheng et al., 2016). IL-6 has been reported to induce dendritic cell (DC) cell maturation (Park et al., 2004) and is related to chemoresistance (Neumann et al., 2018). PDAC is frequently defined as immunologically “cold,” with lower cytotoxic T cell infiltration. Future studies aim to enhance the infiltration of T cells into PDAC tumors and make them “hot” (Pereira et al., 2019). Next, we will summarize the therapeutic targets of CAFs in PDAC and classify them from the main hot spots.

### Targeting the Classical CAF Markers (α-SMA, Fibroblast Activation Protein)

It was believed that as long as fibroblasts were eliminated or inhibited, pancreatic tumor cell growth could be inhibited. Nab-paclitaxel could kill CAFs, thus reducing stromal content and increasing gemcitabine accumulation (Alvarez et al., 2013; Miyashita et al., 2018). However, this hypothesis is under debate due to the lack of therapeutic efficacy (Hidalgo et al., 2015). For a long time, it was customary to think that α-SMA- or fibroblast activation protein (FAP)-positive cells were fibroblasts, which were carcinogenic factors, and studies of therapeutics that targeted α-SMA and FAP have been carried out. Disappointingly, ablation of α-SMA^+^ CAFs caused lower survival rates in an animal model of PDAC due to increased Treg cells and suppressed immune surveillance (Özdemir et al., 2014). FAP in PDAC was highly expressed in CAFs (Aggarwal et al., 2008; Puré and Blomberg, 2018). Various FAP targeting agents, including bispecific antibodies (Wüest et al., 2001; de Sostoa et al., 2019), antibody-drug conjugates (Fang et al., 2016), vaccines (Duperret et al., 2018), and chimeric antigen receptor (CAR) T cells (Lo et al., 2015), all aimed to eliminate FAP^+^ fibroblasts, but anti-FAP therapy resulted in muscle loss, bone toxicities, cachexia and even death (Tran et al., 2013). Targeting α-SMA and FAP has not achieved ideal effects at present, but these preliminary studies also provide a reference for future studies. It has been suggested that depletion of myCAFs leads to the acquisition of EMT, a stem-like phenotype and an undifferentiated state in pancreatic cancer; therefore, inhibition of α-SMA can accelerate tumor progression (Özdemir et al., 2014). FAP^+^ CAFs may be multifunctional mesenchymal stem cells that are recruited into the tumor environment, thus leading to the above malignant results; therefore, the direct inhibition of the FAP^+^ CAF strategy needs to be discussed (Tran et al., 2013).

### Target IL1/LIF/JAK/STAT 3 Pathway

**FIGURE 3:**
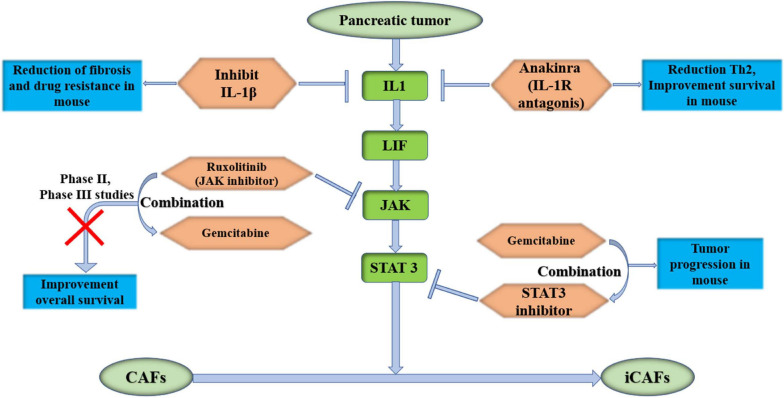
Target IL1/LIF/JAK/STAT 3 pathway, which could activate iCAFs in pancreas. It may become potentially effective targeted therapies for PDAC. IL-1β, interleukin-1β; IL-1R, interleukin-1 receptor; LIF, leukemia inhibitory factor; JAK, Janus kinase; STAT 3, signal transducer and activator of transcription 3; iCAFs, inflammatory cancer-associated fibroblasts.

The STAT3 signaling pathway promotes tumor progression in PDAC (Laklai et al., 2016). Later, Biffi et al. (2019) proved that pancreatic tumor-secreted IL1 activated the LIF/JAK/STAT pathway to activate iCAFs in PDAC to promote tumor growth. This pathway is closely related to iCAFs, and iCAFs are considered to be inflammatory CAFs that promote tumor progression. Therefore, studies of therapies targeting this pathway have been carried out recently. Anakinra, an IL-1R antagonist, which was used to treat IL1^+^ pancreatic cancer tumor cells in mice combined with CAFs, reduced Th2 levels and improved overall survival of pancreatic cancer in a mouse model (Brunetto et al., 2019). IL-1R antagonist-based clinical studies are ongoing (NCT02021422). In another study, Zhang et al. proved that inhibiting IL-1β reduced the fibrosis level of pancreatic tumors and reduced drug resistance in KPC mice (Zhang et al., 2018). Das et al. (2020) found that neutralizing IL-1β with antibodies significantly enhanced the therapeutic efficacy of PD-1 and increased tumor infiltration by CD8^+^ T cells in mice. IL-1β inhibitor-based clinical studies are ongoing (NCT04581343). Targeting IL-1 or its receptors in the presence of CAFs may play a role in future therapies. STAT3 inhibition combined with gemcitabine obviously inhibited tumor growth in a mouse model (Nagathihalli et al., 2015). Ruxolitinib is an inhibitor of the JAK signaling pathway. A phase II study of ruxolitinib combined with capecitabine in clinical therapy has failed in pancreatic cancer. There are some limitations in this study. First, the benefit of ruxolitinib was observed in patients with elevated C-reactive protein (CRP) levels, and modest activity was observed in the intent-to-treat (ITT) population. Second, this was a proof-of-concept study with a limited sample size (Hurwitz et al., 2015). Based on the results of the phase II trial, phase III studies were initiated only in patients with metastatic pancreatic cancer who had failed gemcitabine treatment and had a systemic inflammatory response. Unfortunately, phase III studies have not shown improved overall survival (NCT02119663 and NCT02117479), which suggests that ruxolitinib is an ineffective drug in patients with high levels of CRP (Hurwitz et al., 2018). It is thought that a large number of signaling bypasses and compensatory feedback loops may affect the activity of JAK inhibitors (Hurwitz et al., 2018). Therefore, in the process of CAF activation, it is necessary to further study whether there are bypasses and feedback loops of JAK/STAT3 in the process of CAF activation (Figure 3).

### Target IL-6 and Its Receptor

**FIGURE 4:**
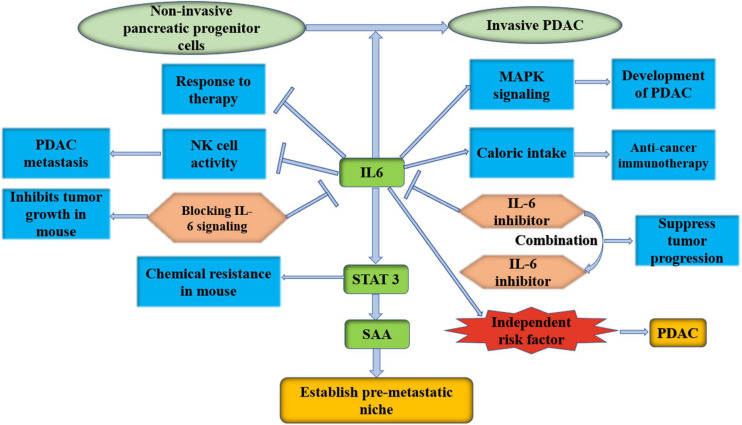
IL-6 is an important marker of iCAFs; it is worth to explore the effect of IL-6 inhibition or its receptor on PDAC progression. IL-6, interleukin-6; PDAC, pancreatic ductal adenocarcinoma; MAPK, mitogen-activated protein kinase; NK cell, natural killer cell; STAT 3, signal transducer and activator of transcription 3; SAA, serum amyloid A.

Inflammatory CAFs show α-SMA^*low*^ IL-6^*high*^ inflammatory feature. CAFs secrete IL-6, which suppresses NK cell activity and leads to PDAC metastasis (Huang et al., 2019). IL-6 promotes the development of PDAC by increasing mitogen-activated protein kinase (MAPK) signaling activation (Zhang et al., 2013). Tumor-induced IL-6 impairs the ketogenic response to inhibit caloric intake, leading to a systemic metabolic stress response that hinders anticancer immunotherapy in PDAC (Flint et al., 2016). A recent study showed that high levels of IL-6 were also related to a reduced response to therapy (Neumann et al., 2018). In addition, it was found that IL-6 secreted by PSCs transformed non-invasive pancreatic progenitor cells into invasive PDAC (Nagathihalli et al., 2016). Blocking IL-6 signaling obviously inhibits primary tumor growth and recurrence in orthotopic xenograft models (Goumas et al., 2015). Mace et al. (2018) inhibited IL-6 and PD-L1 to suppress tumor progression and enhance overall survival in murine models of PDAC, and a combination of targeted therapies that inhibit IL-6 and PD-1 has also recently been launched (NCT04191421). Long et al. (2017) found that IL-6 could activate STAT3 and lead to chemical resistance in PDAC in a mouse model, and the efficacy of chemotherapy in PDAC was improved after the blockade of IL-6 signaling. Higher serum IL-6 was statistically found to be an independent risk factor for PDAC progression to extensive liver metastasis, although it was not associated with longer survival (Kim et al., 2017). During the early stage of pancreatic cancer in mice, liver cells show activation of STAT3 and increased SAA, both of which are dependent on the release of IL-6 into circulation by non-tumor cells. It has also been observed in patients that IL-6-STAT3-SAA signaling promotes the establishment of a prometastatic niche and liver metastasis (Lee et al., 2019). Therefore, inhibition of IL-6-STAT3-SAA signaling can be an effective therapeutic target in the prehepatic niche in PDAC. A clinical trial involving 140 patients with advanced pancreatic cancer was launched, and they were randomly assigned to receive either a chemotherapy plus the IL6 receptor inhibitor tocilizumab or a chemotherapy drug plus a placebo (NCT02767557). As the most important marker of iCAFs, IL-6 plays a very important role. It is worth exploring the effect of IL-6 inhibition or its receptor on tumor progression (Figure 4).

### Target TGF-β and Integrin

Transforming growth factor-β has been shown to reshape CAFs, which induce increased elongation, lamellipodia formation, cell spreading and spheroid invasion in pancreatic cancer (Stylianou et al., 2018). Another study has shown that CAF-secreted TGF-β drives invasive and proliferative phenotypes in PDAC cell lines (Ligorio et al., 2019). Biffi et al. (2019) found that TGF-β promotes the transformation from fibroblasts to myCAFs. Thus, TGF-β has also become a research target for the treatment of PDAC. Early on, TGF-β was targeted in a mouse model, but the result was a deterioration of tumor differentiation and stage (Hezel et al., 2012). Galunisertib is the first oral inhibitor of the TGF-β receptor, and galunisertib combined with gemcitabine improved overall survival in unresectable patients compared with gemcitabine (Melisi et al., 2018). Huang et al. (2019) found that inhibition of the TGF-β receptor reduced IL-6 production from CAFs, leading to decreased STAT3 activation in tumors and reversed immunosuppression in a mouse model. M7824 is a double-fusion protein that blocks both PD-L1 and TGF-β signaling, which inhibits tumorigenesis in mouse models (Lan et al., 2018). Further study should explore whether inhibition of TGF-β signaling in combination with PD-1 inhibitors or gemcitabine can improve the prognosis of pancreatic cancer, and side effects after the combination also need to be avoided.

### Target Shh Signaling

Sonic hedgehog is thought to be highly associated with pancreatic cancer stroma formation. A combination of gemcitabine and the Shh inhibitor IPI-926 has been used in a pancreatic cancer mouse model, and the results showed increased gemcitabine concentrations in the tumor and stabilized the disease (Olive et al., 2009). Another study showed that cyclopamine, a Shh inhibitor, combined with chemotherapy in a mouse model of pancreatic cancer showed prolonged survival (Zhao et al., 2018). However, Shh inhibitors have also been shown to increase tumor growth in mouse models of pancreatic cancer and pancreatic intraepithelial neoplasia (PanIN) progression (Lee et al., 2014; Rhim et al., 2014). In a clinical trial, the Shh inhibitor vismodegib combined with gemcitabine vs. gemcitabine alone did not improve survival after PDAC treatment (Kim et al., 2014). In a randomized Phase Ib/II study of gemcitabine plus vismodegib or placebo to treat metastatic pancreatic cancer, there was no difference in overall response rate, PFS, or OS (NCT01064622) (Catenacci et al., 2015). Phase 2 of this study showed the same result (NCT01088815) (De Jesus-Acosta et al., 2020). A study found that Shh signaling was specifically activated in myCAFs. The smoothened antagonist LDE225 inhibited Shh signaling, reduced myCAF numbers and increased iCAF numbers in mice, which resulted in fibroblast composition and immune infiltration in the PDAC microenvironment (Steele et al., 2021). This study suggests explosive conclusions for the treatment of PDAC and provides great inspiration. Targeting a certain type of CAF specifically for heterogeneity of CAFs, rather than all of the CAFs, may lead to new directions for the treatment of PDAC.

### Target the CXCL12-CXCR4 Axis and CXCL3-CXCR2 Axis

**FIGURE 5:**
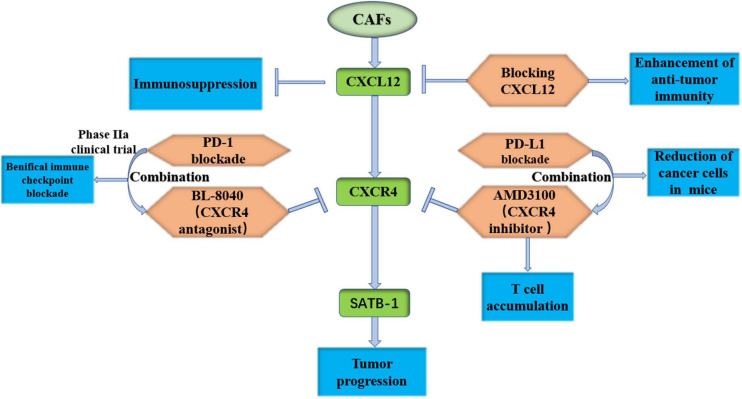
Target the CXCL12-CXCR4 axis is effective in preclinical study. CAFs, cancer-associated fibroblasts; CXCR12, C-X-C motif chemokine receptor 12; CXCR4, C-X-C motif chemokine receptor 4; SATB-1, special AT-rich sequence binding protein 1; PD-1, programmed death 1.

Feig et al. (2013) found that FAP^+^ CAFs secreted CXCL12, which resulted in immunosuppression of PDAC. CXCR4 is the receptor for CXCL12, and an inhibitor of CXCR4, AMD3100, induces T-cell accumulation and works with PD-L1 blockade to reduce cancer cells in KPC mice (Feig et al., 2013). Interestingly, Garg et al. (2018) also found that CXCL12 prevented cytotoxic T-cell infiltration in tumors and killed cancer cells. Blocking the effect of CXCL12 on PDAC cells may enhance antitumor immunity (Garg et al., 2018). It has been found that the SDF-1/CXCR4/special AT-rich sequence binding protein 1 (SATB-1) axis induces malignant progression of PDAC, and inhibition of this axis may be a potential therapeutic target of PDAC (Wei et al., 2018). A phase IIa clinical trial was recently conducted to evaluate the efficacy, safety and immune effects of the CXCR4 antagonist BL-8040 with pembrolizumab and chemotherapy in metastatic PDAC (NCT02826486). The results showed that combined CXCR4 and PD-1 blockade expanded the benefit of chemotherapy in PDAC (Bockorny et al., 2020). We expect more amazing results in an expansion cohort trial combining BL-8040 and panitumumab for second-line treatment (Figure 5). CXCL3 has been shown to be highly expressed in PDAC, and its receptor CXCR2 is almost exclusively expressed in CAFs. CXCL3-CXCR2 signaling could stimulate the transformation of CAFs to myCAFs, which secrete type III collagen and accelerate tumor metastasis (Sun et al., 2021). Another study found that inhibition of CXCR2 reversed tumor progression promoted by type I collagen deletion in a PDAC mouse model (Chen et al., 2021). By summarizing the studies on the CXCL12-CXCR4 axis and CXCL3-CXCR2 axis, two potential inhibition pathways for PDAC are currently effective in preclinical studies. A phase IIa clinical trial showed that combined CXCR4 and PD-1 blockade expands the benefit of chemotherapy in PDAC.

### Target Hyaluronidase

Hyaluronidase (HA) has been shown to be very rich in human PDAC extracellular matrix, which can increase vascular pressure and interstitial fluid pressure, reduce immune cell infiltration and drug penetration, and promote tumor growth (Jacobetz et al., 2013; Wang-Gillam, 2019). Studies have found that patients with low levels of HA live significantly longer than patients with high levels of HA (Whatcott et al., 2015). In a mouse model, an HA inhibitor significantly prolonged survival after combination with gemcitabine (Provenzano et al., 2012). Surprisingly, a phase Ib/II-randomized study of FOLFIRINOX plus pegylated recombinant human hyaluronidase 20 (PEGPH20) versus FOLFIRINOX alone in metastatic PDAC patients showed significant side effects and shortened survival (Ramanathan et al., 2019). Then, a randomized phase III trial of PEGPH20 with nab-paclitaxel plus gemcitabine for patients with HA-high metastatic PDAC was conducted, and it could not improve overall survival or progression-free survival (Van Cutsem et al., 2020). PEGPH20 significantly increased gastrointestinal toxicity and thromboembolic events, increased dose delays and dose reductions, and reduced FOLFIRINOX drug exposure (Ramanathan et al., 2019; Van Cutsem et al., 2020). Therefore, HA inhibition is not recommended because it can induce unexpected clinical effects and serious side effects.

### Target the Acidic Microenvironment or ECM Remodeling

Pancreatic cancer has a dense ECM that insulates T cells and prevents drug penetration. Wang et al. used acidic tumor extracellular pH (PHe)-responsive clustered nanoparticles to deliver drugs in a mouse model (Wang et al., 2020). Wiley et al. (2018) found that CAFs increased the expression of GPR68 (a G-protein-coupled receptor), which could sense acidic environments to further increase fibrosis and IL-6 levels, leading to the development of PDAC proliferation. The acidic environment is closely related to the metabolism of CAFs and accelerates the development of PDAC. Adjusting the pH level of the microenvironment in which CAFs are located, we can explore the communication between CAFs and tumor cells to find targets that could inhibit tumor development. Ji et al. (2017) constructed a tailor-designed matrix metalloproteinase 2 (MMP-2)-responsive amphiphilic peptide and phospholipids, which could release pirfenidone at PDAC and downregulate the many components of the ECM expressed by CAFs. Finally, the penetration of gemcitabine into tumor tissue was significantly increased, and the efficacy of gemcitabine in pancreatic tumors was enhanced (Ji et al., 2017). Diop-Frimpong et al. (2011) found that losartan reduced the generation of type I collagen through CAFs and improved the penetration and therapeutic effect of nanoparticle drugs in tumors. The hexosamine biosynthesis pathway (HBP) is a glycolytic shunt pathway and a metabolic node of cancer cells that can promote survival pathways and influence hyaluronan synthesis in the ECM. Sharma et al. (2020) inhibited this pathway by a small molecule glutamine analog (6-diazo-5-oxo-l-norleucine (DON)). The results showed that DON reduced hyaluronic acid and collagen, leading to extensive remodeling of the ECM, increased CD8^+^ T infiltration, and enhanced sensitivity to PD-1, which ultimately reduced tumor growth and metastasis and prolonged survival. These studies suggest that tumor cells can reprogram the ECM to change the tumor-CAF interaction to promote tumor growth, and the mechanisms by which CAFs reverse ECM remodeling and inhibit tumor development should be explored.

### Others

Targeted therapies for CAFs in pancreatic cancer include focal adhesion kinase (FAK), neuregulin 1 (NRG1), LRRC15^+^ CAFs, etc., The FAK inhibitor suppressed tumor cells and CAFs in a mouse model (Stokes et al., 2011; Kanteti et al., 2018). Jiang et al. (2016) found that the FAK inhibitor VS-4718 was able to respond to immunotherapy and PD1 antagonists by overcoming immunosuppression and fibrosis levels. Inhibition of FAK could reduce ECM remodeling and increase sensitivity to chemotherapy and immunotherapy (NCT02428270, NCT02546531, NCT02651727, and NCT02758587) (Zaghdoudi et al., 2020). NRG1 is secreted by tumors and CAFs, and Ogier et al. (2018) demonstrated that 7E3 (NRG1 antibody) inhibited tumor growth and migration when cocultured with CAFs. LRRC15^+^ CAFs were associated with poor response to anti-PD-L1 treatment (Dominguez et al., 2020). The LRRC15^–^targeted antibody-drug conjugate ABBV-085 demonstrated robust preclinical efficacy (Purcell et al., 2018). Meflin^+^ CAFs inhibited differentiation toward myCAFs and suppressed pancreatic carcinogenesis (Mizutani et al., 2019). Netrin G1 (NetG1)^+^ CAFs supported PDAC survival, and blocking NetG1 inhibited tumor formation *in vivo* (Francescone et al., 2020). These recent studies show that CAFs communicate with pancreatic tumor cells in various ways. At present, research on CAFs has changed from the complete inhibition of CAFs to the intervention of a subpopulation of CAFs, and some initial results have been achieved (Vennin et al., 2019; Table 1).

**TABLE 1:** Clinical trials targeting/relating the CAF in PDAC.

Drug/Target	Mechanism/Strategy	Clinical trial identifies	Phase
Olaptesed pegol (NOX-A12)	CXCL12 inhibitor	NCT03168139 (Completed)	Phase 1/2
Olaptesed pegol (NOX-A12)	CXCL12 inhibitor	NCT03168139(Completed)	Phase 1/2
USL311	CXCR4 inhibitor	NCT02765165 (Terminated)	Phase 1/2
Plerixafor (AMD3100)	CXCR4 antagonist	NCT03277209 (Terminated)	Phase 1
Plerixafor	CXCR4 Antagonist	NCT03277209(Terminated)	Phase 1
Plerixafor	CXCR4 Antagonist	NCT02179970(Completed)	Phase 1
Plerixafor	CXCR4 Antagonist	NCT04177810 (Recruiting)	Phase 2
Motixafortide (BL-8040)	CXCR4 antagonist	NCT02907099 (Active, not recruiting)	Phase 2
VS-4718	FAK Inhibitor	NCT02651727(Terminated)	Phase 1
Defactinib	FAK Inhibitor	NCT02758587 (Recruiting)	Phase 1/2
Defactinib	FAK Inhibitor	NCT03727880 (Recruiting)	Phase 2
Galunisertib	TGF-β Receptor I Kinase Inhibitor	NCT02734160(Completed)	Phase 1
M7824	Bifunctional anti-PD-L1/TGF-β trap	NCT04327986 (Recruiting)	Phase 1/2
M7824	Bifunctional anti-PD-L1/TGF-β trap	NCT03451773(Completed)	Phase 1/2
Canakinumab	IL-1β inhibitor	NCT04581343 (Recruiting)	Phase 1
Anakinra	IL-1R antagonist	NCT02021422 (Unknown)	Phase 1
Anakinra	IL-1R antagonist	NCT02550327 (Active, not recruiting)	Phase 1
Aldesleukin	IL-2	NCT01212887 (Terminated)	Phase 1
Aldesleukin	IL-2	NCT00019084 (Terminated)	Phase 2
BATs Combine With IL-2 and GM-CSF	Anti-CD3 x Anti-EGFR-Bispecific Antibody Armed Activated T-Cells and Low Dose IL-2 and GM-CSF	NCT02620865 (Active, not recruiting)	Phase 1/2
Siltuximab (CNTO 328)	Anti-IL-6 Monoclonal Antibody	NCT00841191 (Completed)	Phase 1/2
Tocilizumab	anti-IL-6-receptor antibody	NCT02767557 (Recruiting)	Phase 2
IL-12	Ad5-yCD/mutTKSR39rep-hIL12 adenovirus	NCT03281382 (recruiting)	Phase 1
PEGPH20	combination with Avelumab	NCT03481920 (Terminated)	Phase 1
PEGPH20	combination chemotherapy (leucovorin calcium, fluorouracil, irinotecan hydrochloride, and oxaliplatin)	NCT01959139 (Active, not recruiting)	Phase 1/2
PEGPH20	combined with nab-paclitaxel plus gemcitabine (PAG treatment)	NCT01839487(Completed)	Phase 2
PEGPH20	combined with nab-paclitaxel plus gemcitabine (PAG treatment)	NCT02715804(Terminated)	Phase 3
Ruxolitinib	JAK signal inhibitor	NCT02119663 (Terminated)	Phase 3
Ruxolitinib	JAK signal inhibitor	NCT02117479 (Terminated)	Phase 3
MK-0646	IGF1R inhibitor	NCT00769483 (Completed)	Phase 1/2
MM-141	IGF1R inhibitor	NCT02399137 (Completed)	Phase 2
Ganitumab	IGF1R inhibitor	NCT01231347 (Terminated)	Phase 3
IPI-926	Hedgehog inhibitor	NCT01383538 (Completed)	Phase 1
VX15/2503	Anti-SEMA4D Monoclonal Antibody	NCT03373188 (recruiting)	Phase 1
zenocutuzumab (MCLA-128)	anti-NRG1	NCT02912949 (recruiting)	Phase 1/2
ADC ABBV-085	an Antibody-Drug Conjugate (ADC) Targeting LRRC15	NCT02565758 (Completed)	Phase 1
Olaratumab	PDGF-α inhibitor	NCT03086369 (Active, not recruiting)	Phase 1/2

*CXCL12, chemokine (C-X-C motif) 12; CXCR4, C-X-C motif chemokine receptor 4; FAK, focal adhesion kinase; TGF-β, transforming growth factor-β; IL-1, interleukin-1; GM-CSF, granulocyte/macrophage colony-stimulating factor; PEGPH20, PEGylated recombinant human hyaluronidase; JAK, Janus kinase; IGF1R, insulin-like growth factor 1 receptor; NRG1, neuregulin 1; LRRC15, 15-leucine repeat membrane protein; PDGF-α, platelet-derived growth factor α.*

## Conclusion

In summary, there are three subtypes of CAFs in pancreatic cancer: iCAFs, myCAFs, and apCAFs. Existing studies have only found their existence and partial functions, but no more evidence links them. A genetic engineering model was used to study pancreatic fibroblast heterogeneity. CAFs also undergo a dynamic process of fibrocyte development, and it is necessary to pay attention to the possibility of type conversion under different tumor microenvironmental states. Therefore, it is necessary to select the appropriate model. Although many studies have found that CAFs are closely related to the occurrence and development of pancreatic malignancies, targeted therapies for CAFs are continuously being carried out, and many targets that are considered to be effective have already failed to achieve the expected effect. The role of CAFs in the pancreas is far more abundant than we think. Although ultimately defined as CAFs, it is possible that different sources may lead to different functions. It is important to trace back to the source of CAFs, which may be the key to opening the door for pancreatic cancer treatment and avoiding many side effects. Finally, it is worth thinking about what kind of conditions in which each type of CAF exists in the body. Could the type of CAF be changed through conditional transformation to improve the microenvironment of PDAC and alleviate tumor progression? Further research is needed to explore the function of each type of CAF and the cross-linking between subtypes of CAFs and other cells. Due to the particularity of CAF enrichment in pancreatic cancer, targeting CAFs is a promising therapeutic direction.

## Author Contributions

XG and HC conceived the presented idea and researched the background of the study. LZ, JH, WY, GL, CC, ZZ, and TZ prepared the figures and tables. XG, HC, LL, and BS wrote the manuscript. All the authors read and approved the final manuscript.

## Conflict of Interest

The authors declare that the research was conducted in the absence of any commercial or financial relationships that could be construed as a potential conflict of interest.

## Abbreviations

α SMA: alpha-smooth muscle actin
Acta2: actin alpha 2
apCAFs: antigen-presenting CAFs
APCs: antigen presenting cells
CAFs: cancer-associated fibroblasts
Col1 α 1: collagen type I alpha 1
Ctgf: connective tissue growth factor
CRP: C-reactive protein
CTLA-4: cytotoxic T-lymphocyte -associated protein 4
CXCL3: chemokine (C-X-C motif) 3
CXCR2: C-X -C motif chemokine receptor 2
DC: dendritic cells
DON: glutamine-fructose amidotransferase 1 (GFAT1) uridine diphosphate *N*-acetylglucosamine (UDP-GlcNAc) 6-diazo-5-oxo-l-norleucine
ECM: extracellular matrix
FAP: fibroblast-activation protein
FAK: focal adhesion kinase
GSEA: enrichment analysis
HAS1: hyaluronic acid synthase 1
HBP: hexosamine biosynthesis pathway
iCAFs: inflammatory CAFs
IFN-γ: interferon γ
IL-6: interleukin-6
IRF5: interferon regulatory factor 5
ITT: intent-to-treat
JAK: Janus kinase
IPMN: intraductal papillary mucinous neoplasm
LIF: leukemia inhibitory factor
LRRC15: 15-leucine repeat membrane protein
MAPK: mitogen-activated protein kinase
MHC II: major histocompatibility complex II
MMP-2: matrix metalloproteinase 2
MTORC1: mTOR complex 1
myCAFs: myofibroblastic CAFs
MYL9: myosin regulatory light chain 9
NetG1: netrin G1
NF- κ B: nuclear factor kappa-B
NRG1: neuregulin 1
PD-1: programmed death 1
PD-L1: programmed cell death-ligand 1
PDAC: pancreatic ductal adenocarcinoma
PEGPH20: PEGylated recombinant human hyaluronidase
PHe: extracellular pH
PSC: pancreatic stellate cell
Saa3: serum amyloid A3
SATB-1: special AT-rich sequence binding protein 1
scRNA-seq: single-cell RNA sequencing
SDF-1: stromal cell-derived factor-1
Shh: sonic hedgehog
SLPI: secretory leukocyte peptidase inhibitor
SMAD2: SMAD family member 2
STAT: signal transducer and activator of transcription
TAGLN: transgelin
TGF-β: transforming growth factor- β
TNF: tumor necrosis factor
TPM1: tropomyosin 1
TWIST1: Twist family BHLH transcription factor 1.
